# Interferon-regulatory factor-1 (IRF1) regulates bevacizumab induced autophagy

**DOI:** 10.18632/oncotarget.5491

**Published:** 2015-09-05

**Authors:** Ji Liang, Yuji Piao, Verlene Henry, Ningyi Tiao, John F. de Groot

**Affiliations:** ^1^ Brain Tumor Center, Department of Neuro-Oncology, The University of Texas, MD Anderson Cancer Center, Houston, TX, USA

**Keywords:** bevacizumab, autophagy, IRF1, antiangiogenesis

## Abstract

**Purpose:**

Antiangiogenic therapy is commonly being used for the treatment of glioblastoma. However, the benefits of angiogenesis inhibitors are typically transient and resistance often develops. Determining the mechanism of treatment failure of the VEGF monoclonal antibody bevacizumab for malignant glioma would provide insight into approaches to overcome therapeutic resistance.

**Experimental Design:**

In this study, we evaluated the effects of bevacizumab on the autophagy of glioma cells and determined target genes involving in the regulation of bevacizumab-induced autophagy.

**Results:**

We demonstrated that bevacizumab treatment increased expression of autophagy markers and autophagosome formation in cell culture experiments as well as in *in vivo* studies. Gene expression profile analysis performed on murine xenograft models of glioblastoma showed increased transcriptional levels of STAT1/IRF1 signaling in bevacizumab resistant tumors compared to control tumors. *In vitro* experiments showed that bevacizumab treatment increased IRF1 expression in a dose and time dependent manner, which was coincident with bevacizumab-mediated autophagy. Down regulation of IRF1 by shRNA blocked autophagy and increased AIF-dependent apoptosis in bevacizumab-treated glioma cells. Consistently, IRF1 depletion increased the efficacy of anti-VEGF therapy in a glioma xenograft model, which was due to less bevacizumab-promoted autophagy and increased apoptosis in tumors with down-regulated IRF1.

**Conclusions:**

These data suggest that IRF1 may regulate bevacizumab-induced autophagy, and may be one important mediator of glioblastoma resistant to bevacizumab.

## INTRODUCTION

Patients with glioblastoma invariably have a short overall survival despite multimodality therapy. This is mainly due to the acquired resistance to standard therapies which leads to tumor recurrence and disease progression [1]. Bevacizumab, a humanized monoclonal antibody that recognizes and blocks vascular endothelial growth factor (VEGF), has been approved for treatment of recurrent glioblastoma [2]. However, the clinical benefits of anti-VEGF therapy in most patients are transient and followed by a restoration of angiogenesis and tumor progression [2, 3]. The mechanisms by which tumors develop resistance desperately need to be identified.

Recent reports suggest that autophagy might play an important role in tumor resistance to anti-VEGF therapy [4-6]. Autophagy is the process by which a cell degrades its own cytoplasmic components and organelles for cell survival. This process involves formation of the autophagosomes. Autophagosomes fuse to the lysosome to produce the autolysosome and enzymatic degradation of autophagosome content to re-usable molecules, which are released back into the cell [7]. This catabolic process is activated in response to specific nutrient deficiencies, general starvation and other cellular stresses [8]. By inhibiting the tumor vasculature, bevacizumab, and other antiangiogenic agents may cause nutrient deprivation and oxygen stress in the tumor microenvironment, both of which are known to induce the process of autophagy. Hu et al. reported that bevacizumab increased regions of hypoxia and higher levels of autophagy-mediating BNIP3 in glioblastoma tumor specimens. *In vivo* targeting of the essential autophagy gene ATG7 in glioblastoma xenograft-bearing mice disrupted tumor growth when combined with bevacizumab treatment [4]. The combined treatment of autophagy inhibitors and bevacizumab increased the efficacy of antiangiogenesis therapy on glioblastoma [4], colon [5] and hepatocellular carcinoma tumors [6]. However, the molecular mechanism by which autophagy confers tumor resistance to bevacizumab is not well understood. The recent study by Li et al. showed that overexpression of interferon-regulatory factor-1 (IRF1) induced autophagy in human hepatocellular carcinoma cells. Silencing IRF1 by small hairpin RNA blocked autophagy induced by interferon-gamma (IFN-γ) [9]. It is unknown if IRF1-regulating autophagy plays a role in glioblastoma resistance to bevacizumab therapy.

In this study, we observed increased IRF1 expression in bevacizumab-resistant tumors, and this expression was parallel with an increase in the molecular hallmarks of autophagy. To determine whether IRF1 plays a role in the regulation of glioblastoma resistance to bevacizumab, glioma cell lines U87 and glioma stem cell-like cells (GSCs) were treated with bevacizumab at different concentrations and the expression of IRF1 was evaluated. We then generated stable glioma cell lines with specific down-regulation of IRF1. These cell lines were implanted into xenograft-bearing mice to determine whether IRF1 impacts the efficacy of bevacizumab therapy in glioblastoma. Our findings demonstrate that bevacizumab-mediated autophagy was decreased in IRF1 down-regulation tumors, which was coincident with increased apoptosis, and an improvement in efficacy of anti-VEGF therapy in glioma xenografts. These data improve the understanding of the mechanisms by which glioblastoma become resistant to bevacizumab and help inform future clinical trials using novel drug combinations.

## RESULTS

### Anti-VEGF treatment increased autophagy in glioma xenograft tumors

**Figure 1:**
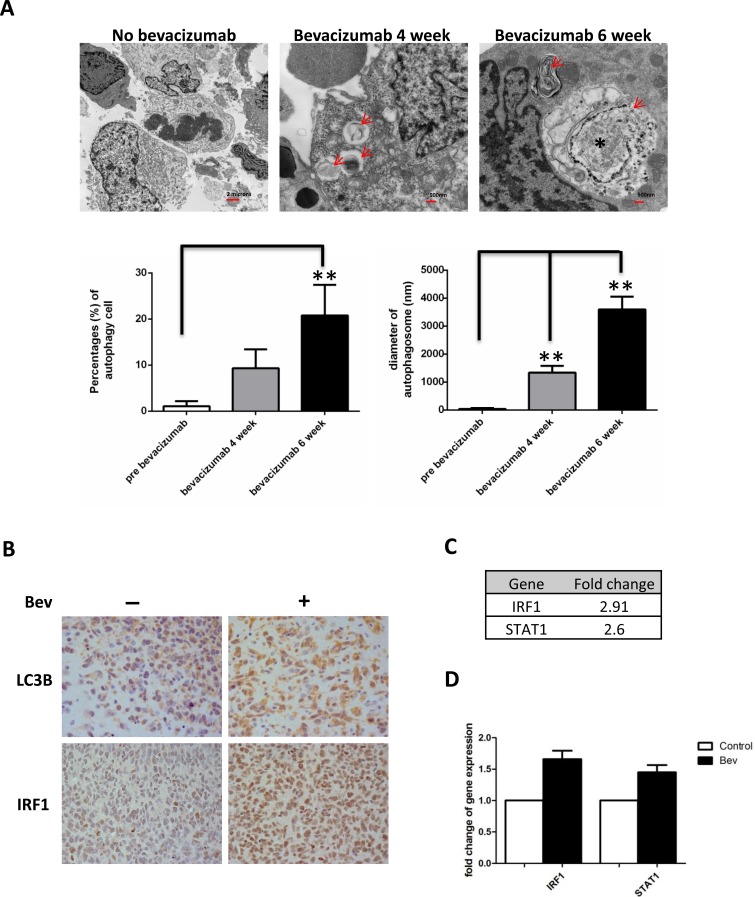
Bevacizumab treatment promotes autophagy in glioma tumors **A.**, autophagy was induced in glioblastoma xenografts in nude mice during bevacizumab therapy. Tumor tissues were collected at 4-6 weeks and analyzed by transmission electron microscopy. Red arrows indicate vacuoles containing multiple membranes and partially degraded material, which are hallmarks of autophagosomes. Asterisks indicate larger vacuoles with degraded material, which likely represent mature autophagic vacuoles. Graphs represent the analysis data of percentages of cells with autophagic vacuoles and diameters of autophagosomes in different groups **: *P* < 0.01, P values were determined by the Student's *t* test. **B.**, bevacizumab treatment increased immunostaining of the autophagy marker LC3 and IRF1 in glioblastoma tumors. (×400 magnification) **C.**, gene chip data showed increased expression of IRF1 and STAT1 in bevacizumab resistant tumors, which was confirmed by real-time PCR analysis **D.**.

Although antiangiogenic therapy is effective in blocking blood vascular and slowing tumor growth, studies in multiple cancer types have shown that tumors eventually acquire resistance to angiogenesis inhibitors [13-15]. Currently, the mechanisms by which this resistance occurs are not well understood. Recently, increasing evidence showed that autophagy might play a role in the tumor resistant to antiangiogenic therapy. In a glioma xenograft mouse model, we found an increased number of cells undergoing autophagy in bevacizumab-treated mice tumors. Tumors treated for 6 weeks displayed a higher number of cells containing autophagosomes (20.77±6.92% vs 1.1±1.14% untreated tumors, *P* < 0.01) but also showed larger autophagosomes (3600±3.39nm, 6 weeks vs 1335.29±1.61nm, 4 weeks vs 35.29±0.36nm untreated tumor, *P* < 0.01) (Figure 1A). Immunohistochemical staining showed increased expression of autophagy marker LC3B in bevacizumab-treated tumors (Figure 1B upper panel). Interestingly, we found bevacizumab increased IRF1 expression in glioma tumors (Figure 1B lower panel). Gene chip analysis of control and bevacizumab resistant glioma tumors also showed up-regulation of STAT1-IRF1 pathway (2.6 fold and 2.91 fold up-regulation for STAT1 and IRF1, respectively, Figure 1C). This result was further confirmed by real-time PCR (Figure 1D).

### Bevacizumab promotes autophagy *in vitro*

**Figure 2:**
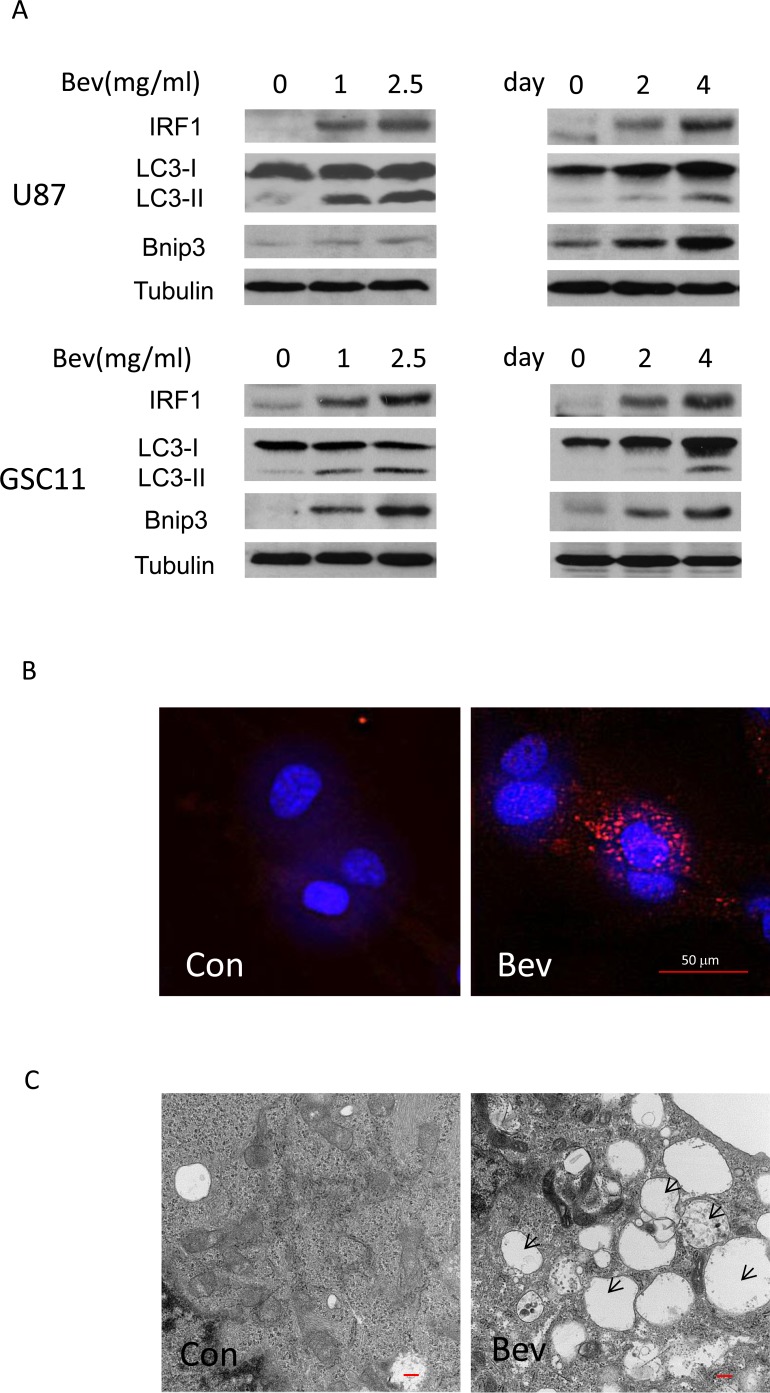
Bevacizumab enhances autophagy *in vitro* **A.**, Western blotting analysis of IRF1, LC3 and Bnip3 in glioma cells treated with the indicated drug concentrations for 72 h or 2.5 mg/ml bevacizumab for the indicated time points. **B.**, representative photo micrographs of LC3 staining (red) of U87 cells incubated in medium containing either buffer (control) or bevacizumab (2.5 mg/ml) for 48 h. Nuclei were stained blue with DAPI. The punctuated distribution of red fluorescence indicated the formation of autophagosomes (Bars, 50 μm) **C.**, TEM. U87 cells were treated as in **B.** and then processed by TEM. Autophagosomes are seen (marked by black arrows) as the rounded vacuolar structures representing characteristics of autophagosomal compartments (Bars, 500 nm).

Angiogenesis inhibition *in vivo* leads to tumor tissue hypoxia and nutrient stress, both of which activate autophagy. To determine whether bevacizumab promotes autophagy independent on oxygen depletion and subsequent hypoxia, two glioma cell lines (U87 glioblastoma cells and GSC11 glioma stem-like cells) were incubated in medium containing either vehicle (control) or bevacizumab. Western blot data demonstrated that bevacizumab induced IRF1 expression, LC3-I to LC3-II conversion and Bnip3 expression in a dose and time-dependent manner (Figure 2A), the latter two are hallmark of autophagy. Similarly, bevacizumab (2.5 mg/ml) significantly increased LC3 punctate staining in U87 cells (Figure 2B and quantified data in Supplemental Figure 1A). Bevacizumab-treated cells showed more autophagosomes; representative electron microphotographs of control and bevacizumab-treated glioma cells are shown in Figure 2C. These finding indicate that bevacizumab promotes autophagy independent of hypoxia.

### IRF1 regulates bevacizumab-promoting autophagy *in vitro*

**Figure 3:**
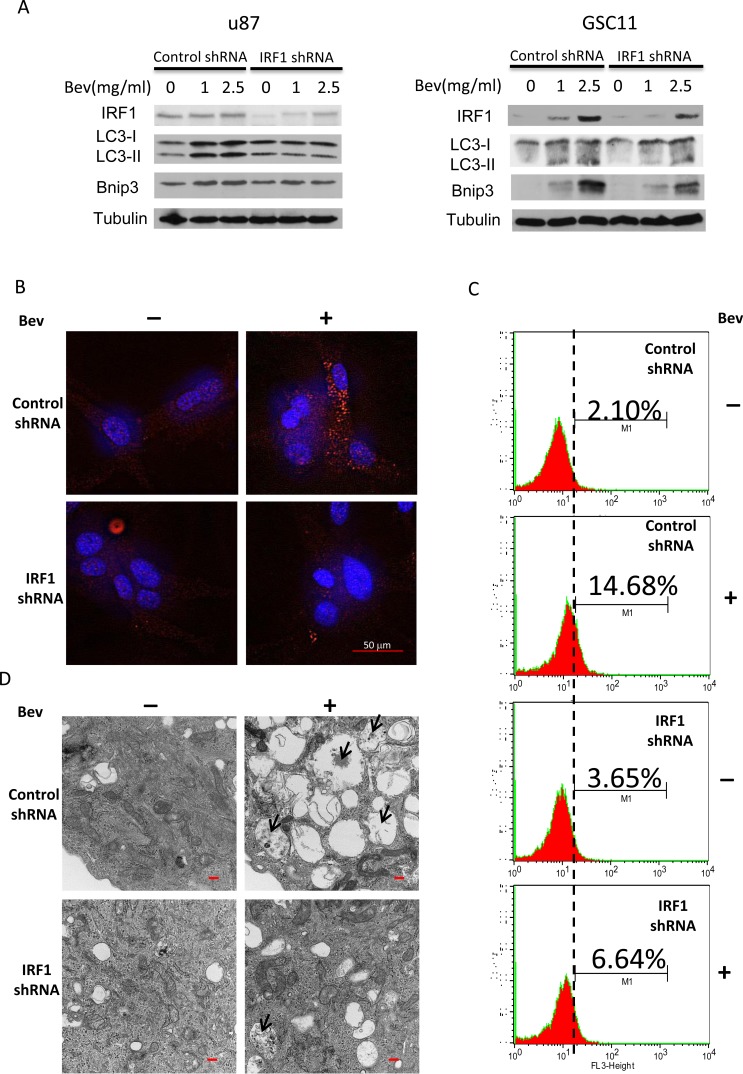
IRF1 depletion blocks bevacizumab-induced autophagy **A.**, Western blot analysis of IRF1, LC3 and Bnip3 in glioma cells stably transfected with control shRNA or IRF1 shRNA. The cells were treated with the indicated drug concentrations for 72 h. **B.**, LC3 immunofluorescence staining. U87 cells transfected with either control shRNA or IRF1 shRNA, were cultured on coverslips and treated with or without 2.5 mg/ml bevacizumab for 48 h. The punctuated LC3 staining (red) was induced by bevacizumab in control cells but less in cells with IRF1 depletion. **C.**, Acridine orange staining. Cells were treated as in **B.** and then stained with 1 μg/ml acridine orange. The indicated numbers represent the differential stainings of acridine orange, which detect the acidic vesicles of autopahgic cells. **D.**, Cells were treated as in **B.** and then processed by TEM. Black arrows point to autophagic vacuoles containing degraded materials (Bars, 500 nm).

We found up-regulation of IRF1 expression concomitant with increased autophagy in bevacizumab-treated gliomas. Recently, a role for IRF1 in the regulation of autophagy has been described [9, 16]. To determine whether IRF1 plays a role in bevacizumab-promoting autophagy, we generated U87 and GSC11 stable cell lines with IRF1 down-regulation. Bevacizumab-induced IRF1 expression in a dose dependent way, which was inhibited by IRF1 specific shRNA (Figure 3A). IRF1 depletion inhibited bevacizumab-induced LC3 conversion and Bnip3 expression (Figure 3A). Similarly, IRF1 depletion reduced LC3 punctate staining (Figure 3B and quantified data in Supplemental Figure 1B) and autophagosomes numbers (Figure 3C) in bevacizumab treated cells. These results were further confirmed using an acridine orange assay, which detects the formation of acidic cellular vesicles, a hallmark of autophagy [17, 18]. GSC11 cells showed an increased percentage of cells stained with acridine orange when treated for 48h with bevacizumab, and this increase was further inhibited by IRF1 depletion (Figure 3D).

### IRF1 depletion increases apoptosis in bevacizumab-treatment glioma cells

**Figure 4:**
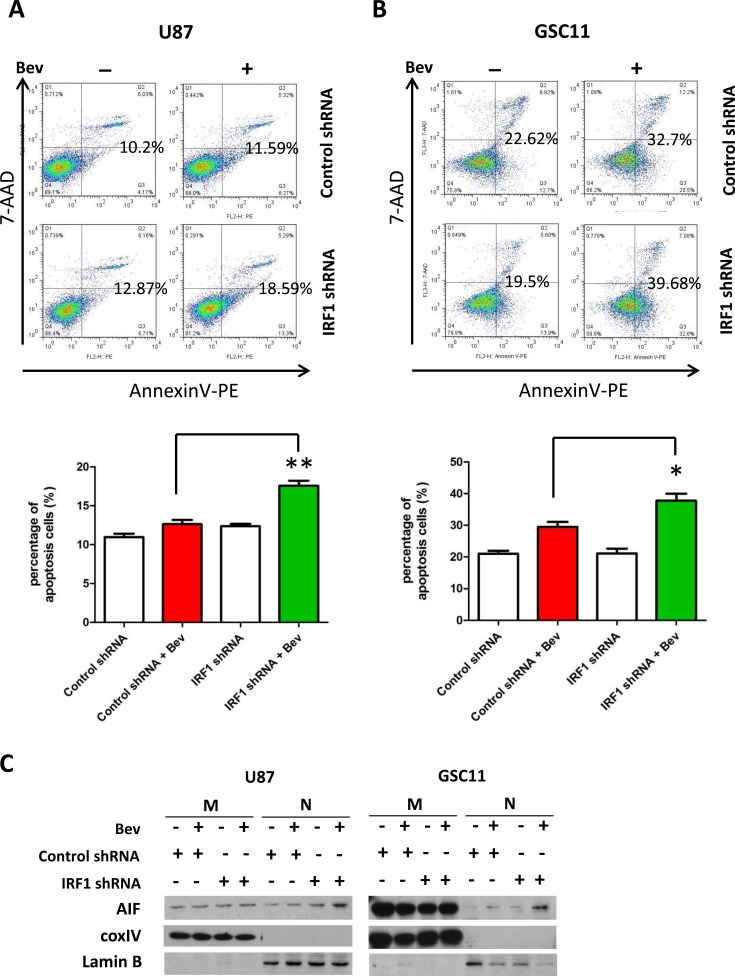
IRF1 depletion increases apoptosis of bevacizumab-treated glioma cells **A.**, control cells or IRF1 down-regulated cells were treated with or without 2.5 mg/ml bevacizumab for 48 h. Apoptotic cells were detected by flow cytometry. The indicated numbers were points to the Annexin V^+^ cells (both early and late apoptosis cells). Graphs represent the percent of apoptotic cells in each group. *: *P* < 0.05, **: *P* < 0.01, *P* values were determined by Student's *t* test. Results are representative of 3 separate independent experiments. C, Western blot analysis of AIF in mitochondrial and nuclear fractions of glioma cells treated as in **A.**. Western blots with coxIV and lamin B-specific antibodies were used to assess protein loading in mitochondrial and nuclear fractions, respectively.

We next analyzed the levels of apoptosis in bevacizumab treated cells. We conducted flow cytometry using PE-Annexin V and 7-AAD to label apoptotic U87 and GSC11 cells (Annexin V^+^ 7-AAD^−^ for early apoptosis and Annexin V^+^ 7-AAD^+^ for late apoptosis). After treatment with bevacizumab for 48 h, the percentage of Annexin V^+^ cells in U87 cells with control shRNA was 12.6±0.7% versus 17.7±0.9% (*P* < 0.01) in U87 with IRF1 shRNA. Similarly, IRF1 down-regulation also increased levels of apoptosis in GSC11 cells treated with bevacizumab (29.47±3.2% in controls compared to 37.77±4.5% in IRF1 knockdown cells, *P* < 0.05). We further found bevacizumab increased expressions of Apoptosis-Inducing Factor (AIF) in nuclear fractions of IRF1 down-regulated U87 and GSC11 cells (Figure 4C), whereas caspase proteins did not change significantly (Supplemental Figure 2). These data suggested that bevacizumab induced AIF-dependent (and caspase-independent) apoptosis in IRF1 down-regulated glioma cells.

### IRF1 depletion increases the efficacy of anti-VEGF therapy in gliomas

**Figure 5:**
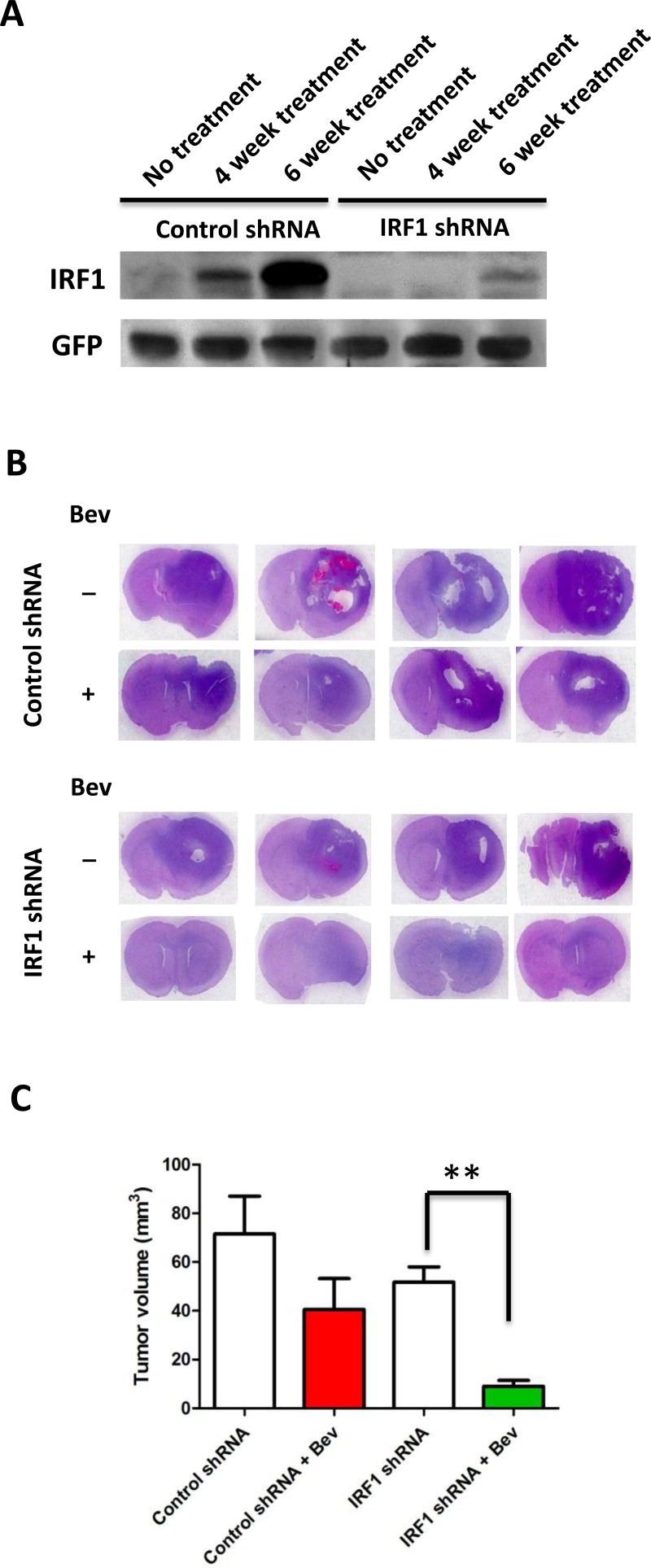
Inhibition of IRF1 increases the efficacy of anti-VEGF therapy in gliomas **A.**, western blot analysis of IRF1 in glioma tumors treated with bevacizumab after 4 and 6 weeks. **B.**, representative whole mounts of H&E stained brains containing GSC11 tumors in nude mice treated with vehicle control or bevacizumab after 5 weeks treatment. **B.**, bar gragh demonstrating the volume of intracranial GSC11 tumors in nude mice (*n* = 7/group). **: *P* < 0.01, *P* values were determined by Student's *t* test.

**Figure 6:**
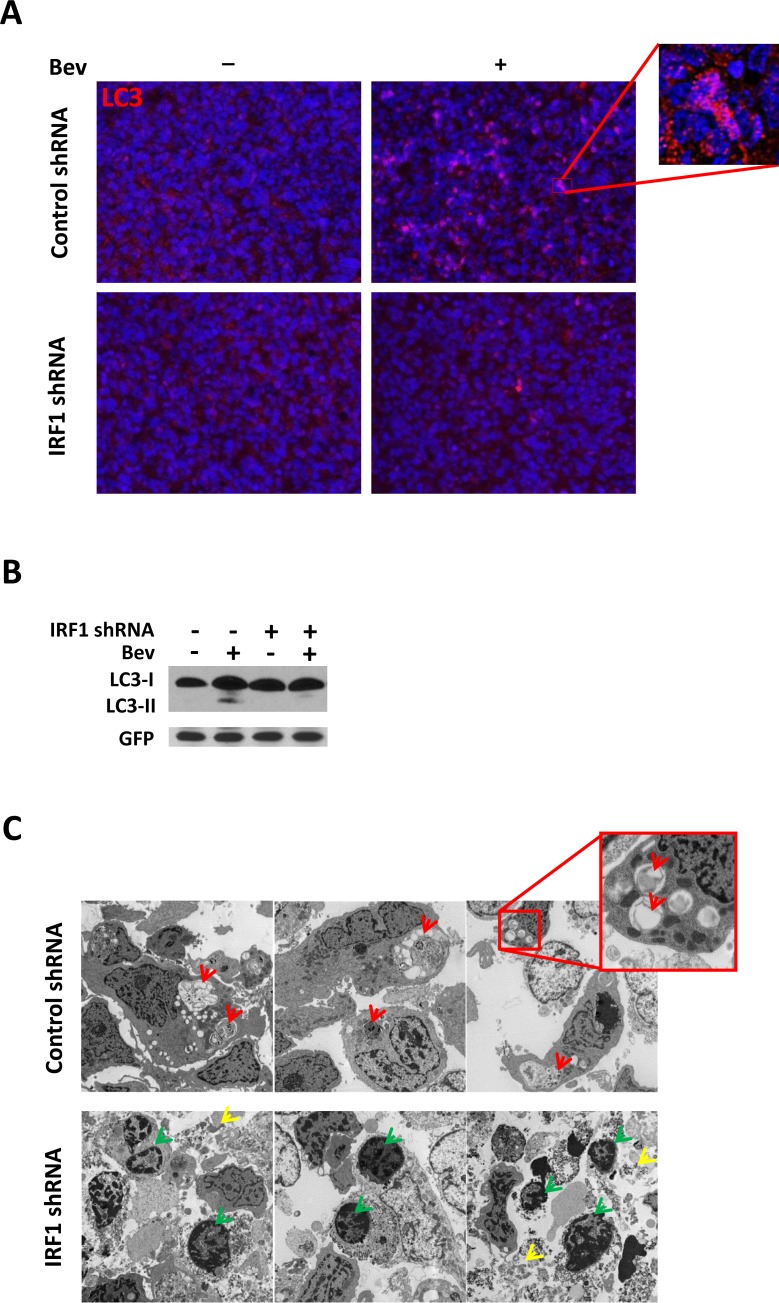

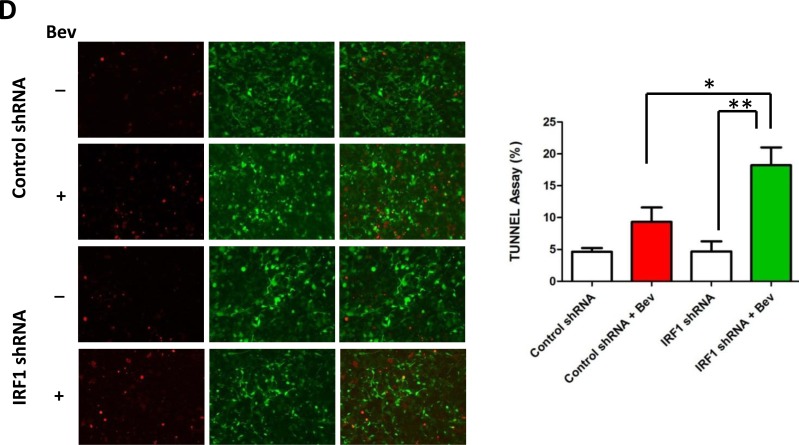
Inhibition of IRF1 leads to decreased autophagy and increased apoptosis in bevacizumab-treated gliomas **A.**, Immunofluorescent staining of LC3 in glioma tumors with or without bevacizumab treatment. (at ×400 magnification) **B.**, Western blot of LC3 conversion in bevacizumab-treated gliomas. Tissue proteins were collected from the tumor part of each brain. GFP-specific antibodies were used to assess protein loading for tumor cells. **C.**, TEM analysis of autophagy and apoptosis in glioma tumors. Red arrows point to autophagic vacuoles while green arrows point to condensed chromatin at the margins of the nucleus, which is the hallmark of apoptosis. Loss of cellular integrity is indicated by yellow arrows. **D.**, TUNEL assay measurement. Glioma tumors from each group were processed using a commercially available TUNEL assay kit. Red fluorescence indicates TUNEL-positive cell, green fluorescence indicates GFP-positive tumor cells. Both TUNEL and GPF positive cells were counted in nine different fields from three individual mice. *: *P* < 0.05, **: *P* < 0.01, *P* values were determined by Student's *t* test.

To determine whether IRF1 plays a role in glioblastoma resistance to antiangiogenic therapy, IRF1 down-regulated GSC11 cells were implanted into nude mice. The mice were euthanized at different time points and their brains removed and processed for analysis. Western blot analysis showed that the expression of IRF1 increased during bevacizumab treatment that was not visible in IRF1 down-regulated mice tissues (Figure 5A). Figure 5B showed the representative images of 5 week treated mice brain H&E staining. Bevacizumab significantly decreased tumor volume in IRF1 down-regulated tumors (Figure 5C, 46.6±6.9 vs 9.0±2.6, *P* < 0.01), whereas tumor volume was not decreased by bevacizumab in control shRNA tumors (71.5±16.8 vs 42.0±11.1, *P* = 0.18, Figure 5C). Since IRF1 regulated autophagy in glioma cells *in vitro*, we then evaluated autophagy level in glioma xenograft tumor tissues. We found bevacizumab increased the autophagy marker LC3 staining in control shRNA tumors, which was not observed in IRF1 shRNA tumors (Figure 6A). Western blot analysis also showed increased LC3 expression and LC3-I to LC3-II conversion in bevacizumab-treated control tumors, but not in IRF1 shRNA tumors (Figure 6B). Transmission electron microscope images of 6-week treated tumor tissues showed that autophagosomes (Figure 6C, upper panel, red arrows) were easily found in bevacizumab-treated control tumors. In IRF1 down-regulated tumors, instead of this hallmark of autophagy, tumors displayed condensed chromatin at the margins of the nucleus (Figure 6C, lower panel, green arrows) and increased cells with loss of cellular integrity (Figure 6C, lower panel, yellow arrows), which are classic signs of an apoptotic process. Consistently, TUNEL assay showed that more apoptotic signals were observed in IRF1 down-regulation tumors, compared to control tumors treated with bevacizumab (Figure 6D, *P* < 0.01). Taken together, these data indicate that IRF1-regulated autophagy might be one mechanism by which glioma tumors escape from the antiangiogenic therapy. Inhibiting this autophagic progress might promote apoptosis in glioma tumor cells and increase the efficacy of antiangiogenic therapy in gliomas.

## DISCUSSION

The proposed efficacy of antiangiogenic therapy stems from its ability to devascularize a tumor and thereby limit tumor growth. However, the efficacy of antiangiogenic therapy is limited as malignant gliomas appear to have high levels of intrinsic and acquired resistance leading to short-lasting benefits of this therapeutic approach [13, 19]. The understanding of resistance mechanisms remains a major limitation towards improving outcomes for patients. Although experimental evidence demonstrates that infiltration and recruitment of proangiogenic cells contributes to adaptive resistant to antiangiogenic therapy [20, 21], more and more data demonstrate that “tumor escape” from antiangiogenic therapy is a complicated process that involves multiple mechanisms [22]. The major aim of this study was to determine the possible mechanisms underlying glioblastoma resistance to bevacizumab, which is the most commonly used angiogenesis inhibitor. We compared gene expression profiles between control and bevacizumab resistant glioblastoma tissues. Our data showed bevacizumab treatment increased the expression of several genes including the transcription factor-IRF1. We further found IRF1-regulated autophagy promoted glioblastoma resistance to bevacizumab treatment. Inhibition of this IRF-1-mediated autophagy led to increased apoptosis and enhanced efficacy of anti-VEGF therapy.

IRF1 was the first of 9 identified mammalian members of the IRF family [23, 24]. When induced by various stimuli, such as viral infection and interferon, IRF1 acts as a transcriptional activator of specific IFN-stimulated genes that mediate diverse functions including cellular responses to inflammation, programmed cell death and tumor suppression [23, 25-28]. Recent studies reported IRF1 also participates in regulating autophagy with growth inhibition and cell death. Silencing IRF1 expression blocked autophagy induced by IFNγ [9]. However, another recent study demonstrated increased autophagy and decreased apoptosis in IRF1 knockout mice in a murine endotoxemia model [29]. In our study, we found IRF1 down-regulation decreased autophagy and increased apoptosis in bevacizumab-treated glioblastoma. These disparate results may be attributed to the different models that lead to different posttranslational modification of IRF1, such as SUMOylation and phosphorylation [30-33]. Park et al. reported that inoculation of SUMOylated IRF1-transfected cells into athymic nude mice resulted in tumor formation, suggesting SUMOylation converted the tumor suppressor IRF1 into an oncogenic protein [33]. This function-converted modification was also observed in the regulation of STAT1, which is an upstream regulator of IRF1. A recent study by Zimmerman at al. showed that phosphorylation status of pSTAT1 determines its function as a tumor suppressor, with unphosphorylated STAT1 acting as a tumor promoter that acts by elevating resistance to Fas-mediated apoptosis to promote immune escape [34]. These data demonstrate that the STAT1-IRF1 pathway can be regulated by complicated posttranslational modifications that confer proteins to exert opposite roles. Our *in vitro* experiments on glioma cells showed that bevacizumab not only increased the expression level of IRF1, but also increased the size of IRF1 (Figure 2A and 3A), suggesting a possible posttranslational modification by bevacizumab treatment. It is possible that posttranslational modification of IRF1 is involved in bevacizumab-induced autophagy, which partly contributes to glioblastoma resistance to this therapy. Further studies are needed to identify the molecular modulation leading to posttranslational changes in IRF1 following drug treatment.

Recent evidence suggests that although autophagy may initially prevent tumor formation and growth, tumor cells respond to treatment-related stressors by using autophagy as a cyto-protective mechanism leading to treatment resistance [4-6]. The devascularization caused by bevacizumab increases tumor hypoxia and nutrient deprivation in xenograft animal models. *In vitro* experiments have shown that both hypoxia [5, 35] and nutrient starvation [6] induce autophagy. Interestingly, our data showed that bevacizumab can directly induce autophagy *in vitro*, independent of tumor microenvironment effects. Therefore, besides oxygen stress and nutrient deprivation, direct sequestration of VEGF with bevacizumab may directly promote autophagy, which leads to drug resistance during anti-VEGF therapy. The direct role of VEGF in autocrine and paracrine signaling in glioma and its impact on cell survival mechanisms deserves additional study.

Our *in vitro* data demonstrate IRF1 as a dual regulator of autophagy and apoptosis in gliomal cell lines with bevacizumab treatment. This phenomenon is also observed in xenograft animal models and it has been shown to contribute to adaptive resistance to bevacizumab therapy. However, we cannot exclude that IRF1 might play additional roles in the regulation of glioblatoma resistance to bevacizumab considering the heterogeneity of the tumor microenvironment *in vivo*. For example, STAT1-IRF1 pathway might affect tumor progression and patients outcome by regulating infiltrations of immune cells [37]. More investigations of IRF1 function *in vivo* should be explored in the future.

Currently, chloroquine (CQ) and its derivatives are the only inhibitors of autophagy that are available for use in the clinic. Some studies have shown that although CQ might be beneficial when used in combination with cancer therapy drugs, its sensitizing effects can occur independently of autophagy inhibition [38, 39]. More specific autophagy inhibitors are clearly needed. Here we provided evidence that IRF1 may play a role involved in autophagy regulation and the interplay between autophagy and apoptosis in the setting of VEGF inhibition. Combined treatment of an IRF1 inhibitor and bevacizumab may increase the efficacy of antiangiogenic therapy on glioblastoma.

## MATERIALS AND METHODS

### Cell culture and transfection

Glioma stem cell-like cell line GSC11 was derived from a glioblastoma patient that did not receive treatment with bevacizumab and has been previously described [10]. Human glioblastoma cell line U87 was obtained from American Type Culture Collection. U87 cells were maintained in DMEM containing 10% fetal bovine serum, and GSC11 cells were maintained in suspension in DMEM containing epidermal growth factor, basic fibroblast growth factor (bFGF), and B27 (Invitrogen) at 37°C in 5% CO_2_ atmosphere. For transfection, cells were plated at a density of 3×10^5^/6 well plate 3 h prior to transfection. Transfection was carried out using HyFect reagents according to the vendor's instructions. Transfected cultures were selected with puromycin (5 μg/ml) for 10-14 days. At that time, antibiotic-resistant colonies were picked, pooled and expanded for further analysis under selective conditions. The pGIPZ control was generated with control oligonucleotide GCTTCTAACACCGGAGGTCTT. pGIPZ IRF1 shRNA was generated with TAGTGTACACCTCTGATCA. For cell treatment, human lgG was used as a control for all experiments, and bevacizumab was added at the concentrations indicated [11].

### Immunohistochemistry

Paraffin sections from xenografts were used for immunohistochemical analysis. The slides were deparaffinized and subjected to graded rehydration. After blocking in 5% serum and an antigen retrieval step (citrate buffer, pH 6.0), slides were incubated with primary antibodies to CD31 (1:50, Abcam), LC3B (1:50, CST), IRF1 (1:50, Santa Cruz) overnight at 4°C. After washing in PBS with Tween 20, primary antibody reactions were detected using the Vectastain ABC kit (Vector Laboratories) with the respective secondary antibody.

### Real-time polymerase chain reaction (PCR)

Total RNA was extracted from tumor bearing mouse brain tissue using RNeasy Mini Kit coupled with DNase treatment (QIAGEN) and reverse transcribed with High Capacity cDNA Reverse Transcription Kit (Applied Biosystems). Each cDNA was analyzed in triplicate using real-time TaqMan probes (Applied Biosystems). Quantitative PCR analysis was performed using a chromo 4 sequence-detection system (Bio-Rad, Hercules, CA, USA). Relative quantification of mRNA levels was performed using the comparative CT method with GADPH as the reference gene and the formula 2 ^−ΔΔc^_t_.

### Immunoblot analysis

Cells were lysed in an ice-cold lysis buffer containing 50 mM Tris-Cl, pH 7.5, 100 mM NaCl, 1 mM EDTA, 1% TritonX-100, 1 mM PMSF, 1 μg/ml leupeptin, and 1 μg/ml pepstain A. The protein concentration in the supernatant was determined using a BCA protein assay (Pierce, Rockford, IL, USA). Samples were subjected to 8-12% SDS-polyacrylamide gel electrophoresis, and the separated proteins were electrophoretically transferred to nitrocellulose membranes. Blots were incubated with the primary antibody against IRF1 (1: 1000, Santa Cruz Biotechnology), LC3B (1:1000, CST), Bnip3 (1:1000, CST), Tubulin (1:3000, Sigma), AIF (1: 1000, CST), coxIV (1: 1000, Calbiocam), Laminin B (1: 1000, Abcam). The membranes were then incubated with horseradish peroxidase-linked secondary anti-rabbit or anti-mouse antibodies (Bio-Rad).

### Immunofluorescence staining

Immunofluorescence analysis was performed as previously described with minor modifications [12]. Briefly, formaldehyde-fixed cells were permeabilized with Triton X-100 0.1% in PBS, and blocked with 5% serum diluted in PBS-gel (0.2% gelatin in PBS) for 30 min. The primary antibodies were incubated in blocking solution overnight at 4°C. Immuno-staining was performed using the primary antibody against LC3B (1:50, CST). Coverslips were mounted using ProLong antifade reagent (Invitrogen). The images were acquired with an inverted deconvolution microscope. Images were taken with a Zeiss Axioskop 40 microscope equipped with AxioVision Rel.4.2 software.

### Transmission electron microscopy (TEM)

Samples were fixed with a solution containing 3% glutaraldehyde plus 2% paraformaldehyde in 0.1 M cacodylate buffer, pH 7.3, for 1 hour. After fixation, the samples were washed and treated with 0.1% Millipore-filtered cacodylate buffered tannic acid, postfixed with 1% buffered osmium tetroxide for 30 min, and stained en bloc with 1% Millipore-filtered uranyl acetate. The samples were dehydrated in increasing concentrations of ethanol, infiltrated, and embedded in LX-112 medium. The samples were polymerized in a 60°C oven for 2 days. Ultrathin sections were cut in a Leica Ultracut microtome (Leica, Deerfield, IL), stained with uranyl acetate and lead citrate in a Leica EM Stainer, and examined in a JEM 1010 transmission electron microscope (JEOL, USA, Inc., Peabody, MA) at an accelerating voltage of 80 kV. Digital images were obtained using AMT Imaging System (Advanced Microscopy Techniques Corp, Danvers, MA).

### Acridine orange staining

To detect the formation of acidic vesicular organelles, cells were treated and then stained with 1.0 Ag/mL acridine orange for 15 min. Cells were then trypsinized, collected in PBS, and analyzed using a FACScan flow cytometer.

### Apoptosis assays

Apoptosis was quantified using Annexin V-PE 7AAD Apoptosis Detection kit I (BD) according to the manufacturer's instructions. U87 and GSC11 cells were incubated in standard culture conditions with and without drug treatment for 72 h before staining and subsequent flow cytometric analysis. We slso analyzed tumor tissue for evidence of apoptosis using a terminal deoxynucleotidyl transferase-mediated dUTP nick-end labeling kit per the manufacturer's instructions (Roche).

### Animal xenografts

For *in vivo* experiments, GSC cells (3 × 10^5^) were implanted intracranially into nude mice (7-9 mice per group). Beginning 4 days after implantation, bevacizumab (10mg/kg) or vehicle was administered by i.p. injection twice a week [12]. The mice were euthanized at 4 and 6 week, and their brains were removed and processed for analysis. All experiments were approved by the Institutional Animal Care and Use Committee of The University of Texas M. D. Anderson Cancer Center. Animal survival analysis was performed using the Kaplan-Meier method and tumor volume comparisons between groups were compared using the log-rank test. *P* < 0.05 was determined to be significant.

## SUPPLEMENTARY MATERIAL FIGURES


