# Supplementary material for: Interferon-regulatory factor-1 (IRF1) regulates bevacizumab induced autophagy
Source: Oncotarget. 2015 Sep 5;6(31):31479–92. doi: 10.18632/oncotarget.5491 (PMC4741619; doi:10.18632/oncotarget.5491)
Supplement: Supplementary file 1 [file oncotarget-06-31479-s001.pdf]

Interferon-regulatory factor-1 (IRF1) regulates bevacizumab induced autophagy

Supplementary Material

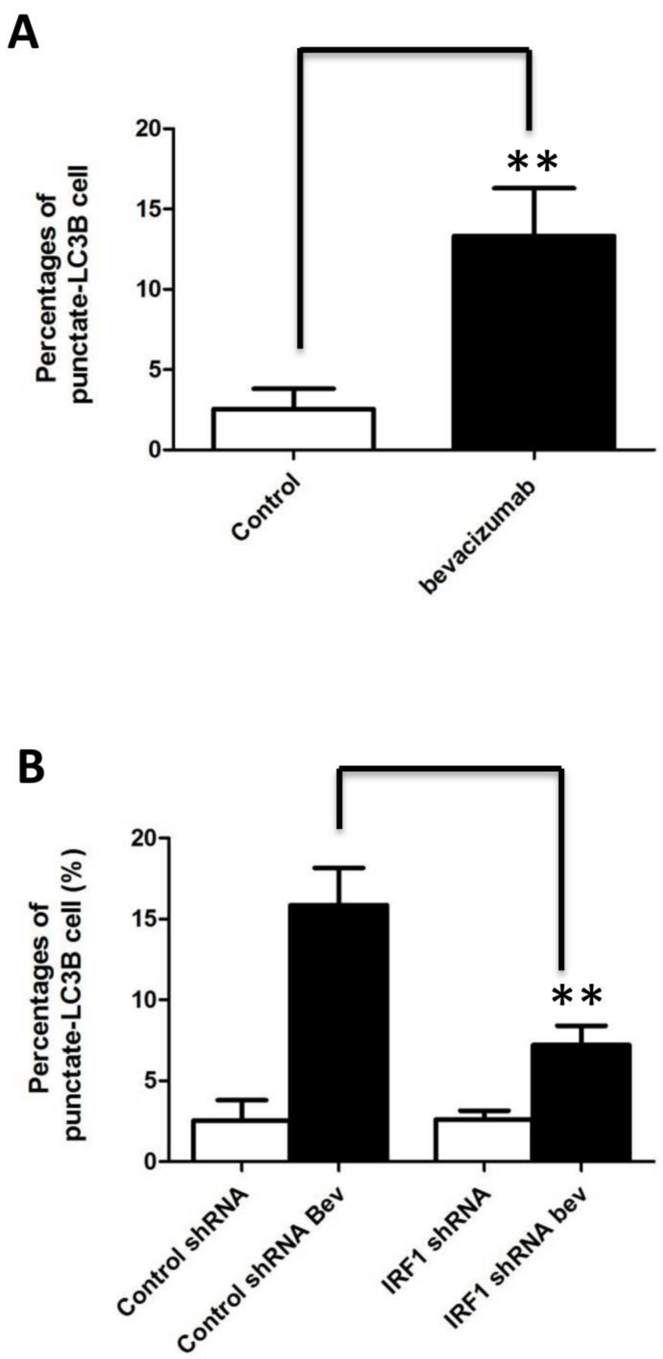

Supplemental Figure 1: Quantified data of LC3 immunofluorescence staining. A., The punctuated LC3 staining was increased by bevacizumab. **B.**, IRF1 depletion reduced bevacizumab-induced LC3 punctate staining. **: $P < 0.01$, P values were determined by Student's t test.

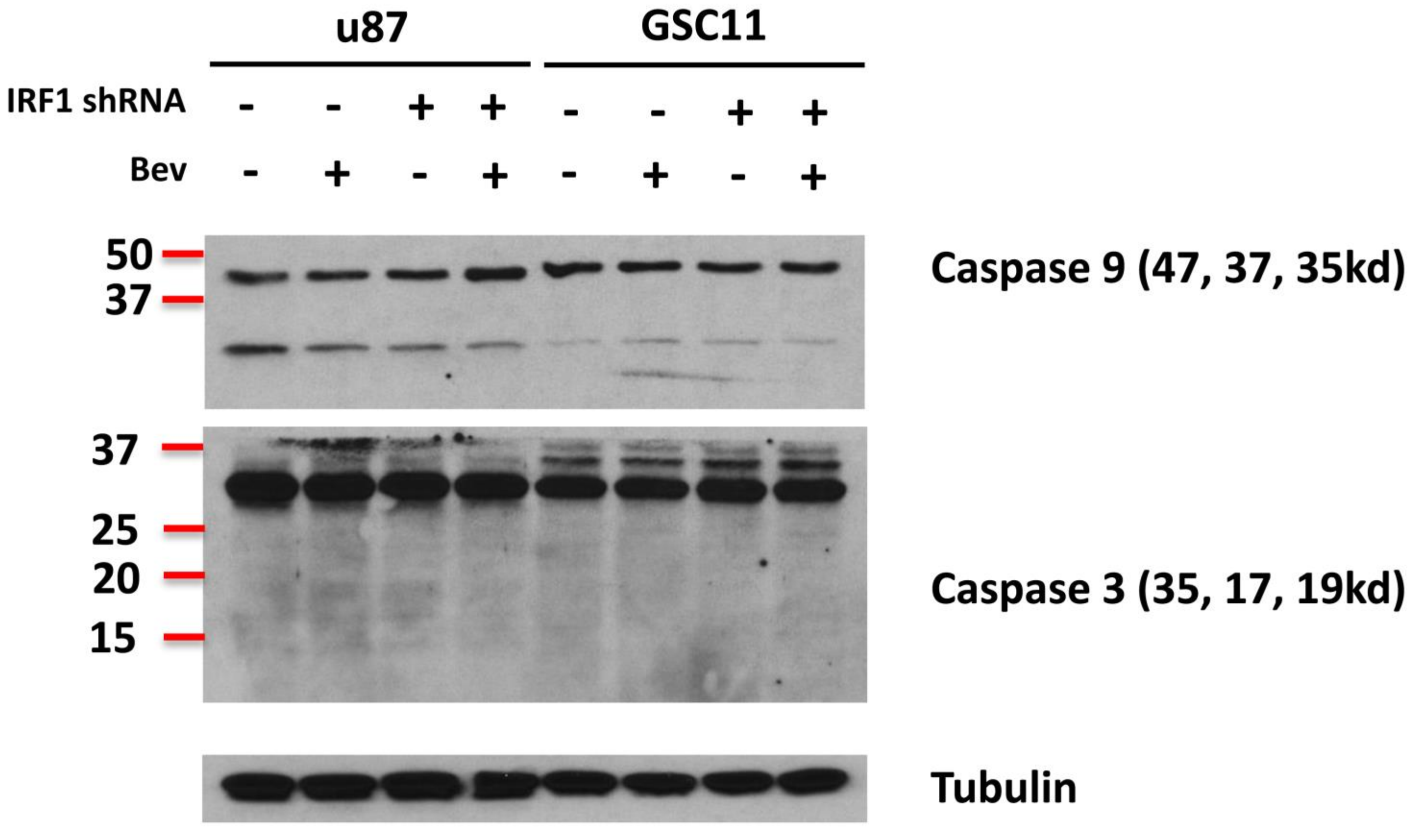

Supplemental Figure 2: Bevacizumab and IRF1 depletion do not affect the expressions of caspase 3 and caspase 9.
